# Webcast marketing platform optimization via 6G R&D and the impact on brand content creation

**DOI:** 10.1371/journal.pone.0292394

**Published:** 2023-10-19

**Authors:** Hui Wen

**Affiliations:** School of Management, Henan Institute of Economics and Trade, Zhengzhou, Henan, China; UTM Skudai: Universiti Teknologi Malaysia, MALAYSIA

## Abstract

This work aims to investigate the development and management of cosmetics webcast marketing platforms, offering novel approaches for building and sustaining commercial brands. Firstly, an analysis of the current utilization of cosmetics webcast marketing platforms is conducted, identifying operational challenges associated with these platforms. Secondly, optimization strategies are proposed to address the identified issues by leveraging advancements in 6th Generation (6G) communication technology. Subsequently, a conceptual framework is established, employing big data interaction to examine the influence of webcast marketing platform experiences on brand fit. Multiple hypotheses are formulated to explore the relationship between platform experiences and brand fit. Finally, empirical analysis is performed within the context of the 5th Generation (5G) Mobile Communication Technology and extended to incorporate the 6G Mobile Communication Technology landscape. The results of the validation indicate the following: (1) the content generated by the webcast marketing platform has a positive impact on brand fit (β = 0.46, p<0.01; β = 0.31, p<0.05); (2) in the 6G network environment, a webcast marketing platform with high traffic transmission rates may enhance brand fit (β = 0.51, p<0.001); (3) the content generated by the webcast marketing platform exhibits significant positive regulatory effects on information-based and co-generated content (β = 0.42, p<0.01; β = 0.02, p<0.001). The findings of this work offer valuable insights for other scholars and researchers seeking to optimize webcast marketing platforms.

## Introduction

In the digital era, the field of marketing is undergoing an unprecedented transformation. With the continuous advancement of technology and the rapid development of the Internet, webcast marketing has emerged as a crucial tool for brand promotion and consumer interaction [1, 2]. However, with the imminent arrival of the 6th Generation (6G) Mobile Communication Technology, webcast marketing platforms are on the brink of a revolutionary change. This work delves into the optimization of webcast marketing platforms based on 6G research and examines its significant impact on brand content creation. In today’s mobile internet-driven landscape, the creation of compelling brand content has become a vital differentiating factor for companies striving to excel in a fiercely competitive market. Webcast marketing platforms offer brands the opportunity for direct engagement with consumers through real-time video transmission and interactive features, fostering a deeper connection. Nonetheless, traditional webcast technologies have inherent limitations concerning bandwidth, latency, and interactivity, which hinder brands’ content creation capabilities. The introduction of 6G technology heralds a comprehensive upgrade for webcast marketing platforms. The ultra-high-speed transmission, low latency, and ample capacity features of 6G facilitate smoother, more transparent, and more interactive webcast experiences, presenting immense opportunities for brand content creation. Brands can harness high-quality videos, virtual reality, and augmented reality technologies enabled by 6G to create immersive content experiences that captivate consumer interest and forge emotional connections. Concurrently, the high bandwidth of 6G supports expanded data transmission and cloud services, granting brands more excellent creative freedom and personalized customization capabilities.

In today’s fiercely competitive market environment, the efficacy and innovation of brand communication are integral determinants of business success [3]. Webcast marketing, characterized by its intuitive and highly interactive nature, not only facilitates the transmission of more authentic and vivid brand messages but also enables real-time engagement with the audience, thereby fortifying the emotional bond between brands and consumers. However, technological support and platform optimization are indispensable prerequisites to achieving effective webcast marketing beyond the provision of high-quality content and engaging interactive approaches. As the forthcoming generation of mobile communication technology, 6G technology is poised to deliver exceptional communication capabilities that promise enhanced performance and superior user experiences for webcast marketing platforms. Therefore, the primary objective of this work is to conduct an extensive investigation into the methodologies and outcomes of harnessing 6G technology to augment webcast marketing platforms while also exploring the resulting impact on brand content creation [4].

This work aims to comprehensively analyze the favorable outcomes of optimizing webcast marketing platforms through 6G technology advancement, particularly in relation to brand content creation. This investigation takes into account various dimensions encompassing technical performance, user experience, and the effectiveness of brand communication. By delving into the innovative applications of live streaming platforms within the purview of 6G technology, our intention is to furnish tailored strategies for businesses and marketers, thereby facilitating the attainment of their brand communication and marketing objectives more effectively. In essence, this work seeks to contribute valuable research insights and pragmatic implications to both academic and practical domains. By exploring the optimization of webcast marketing platforms within the framework of 6G technology and its consequential impact on brand content creation, our endeavor aspires to provide a significant resource for the progress of webcast marketing in the context of emerging technologies.

## Literature review

### Webcast marketing

In recent years, the rise of the online webcast economy has introduced a new marketing model into people’s lives, with companies recognizing online webcast platforms as valuable frontiers for marketing activities. Within the field of webcast marketing, extensive research has been conducted. Zhao et al. (2022) emphasized the growing significance of hosts in webcast marketing models compared to traditional marketing approaches, highlighting them as a research focus. Their study investigated the effects of different product types and host styles on consumer psychology and behavior. The findings revealed that (1) for functional products, the entertainment host style significantly increased consumers’ emotional identification but reduced their perception of authority and knowledge, and (2) for functional products, the knowledge anchor style heightened consumers’ perception of authority and knowledge while diminishing emotional identification [5]. Liao and Chiu (2021) [6] highlighted the popularity of mobile games as a form of online leisure and communication, attracting players of various age groups and expanding the market. Online streaming refers to real-time entertainment and communication activities conducted through the Internet. The authors conducted a market survey of Taiwanese manufacturers and analyzed 1020 questionnaire responses in a relational database to discern the profiles, preference patterns, and behavior rules of game players. Through cluster analysis, they classified Taiwanese mobile game players into three groups and identified the characteristics of each group. Additionally, the authors devised a rule-based recommendation method, association rules, to examine the online streaming and purchasing behavior of mobile game players from the perspective of webcast marketing recommendations. Shutaleva et al. (2022) [7] observed the flourishing trend of marketing methods employing social media as a conduit, driven by ongoing advancements in science and technology. In light of this backdrop, the authors employed a combination of theoretical research and empirical analysis. They selected three models of webcast marketing: “brand + celebrity + webcast,” “brand + amateur + webcast,” and “brand + press conference + webcast.” These models were integrated with corporate brand images, with brand identity introduced as a mediating variable to examine the impact of webcast marketing on corporate brand images. The authors identified key factors associated with webcast marketing models that influence corporate brand images. Rakshit et al. (2022) [8] acknowledged that the development of Internet technology had provided a foundation for the growth of webcast platforms. Prominent platforms like YY Live have become significant venues and channels for brand marketing. Building upon theories related to interactive marketing in the webcast, the authors analyzed interactive marketing case studies involving notable mobile phone brands on the YY Live platform. They conducted an in-depth investigation into the current state of interactive marketing by major mobile phone brands on the YY Live platform. This work revealed that most companies perceive webcast as a one-way platform for disseminating information about their mobile products, lacking effective interactive strategies and a marketing mindset that integrates live interactive marketing activities with the essence of mobile phone brands. YY Live has not fully harnessed the unique advantages of interactive marketing in the realm of mobile phone brand marketing. Qing et al. (2021) [9] highlighted webcast as a product of the mobile Internet era, with the development of mobile technology and the marketing industry’s needs propelling webcast marketing into a standard practice for businesses. The authors approached their study from the perspective of problem discovery, analysis, and solution, aiming to analyze and establish a standardized advertising model for cosmetics in webcast marketing to ensure its sustainable development.

In conclusion, notable progress has been achieved in webcast marketing research; however, several limitations persist. The following key issues within the field of webcast marketing research have been identified:

1. Incomplete theoretical framework: The existing theoretical research on webcast marketing is relatively limited, and the available frameworks lack comprehensiveness. There is a dearth of in-depth exploration regarding the characteristics, influencing factors, and effectiveness evaluation of webcast marketing, resulting in a relatively weak theoretical foundation in this domain.
2. Insufficient methodology: The research methods utilized in webcast marketing are still relatively constrained. Many studies heavily rely on questionnaire surveys and case analyses, which lack more systematic and comprehensive research methods and robust data analysis techniques. Conducting additional empirical research and employing quantitative analysis are necessary to enhance the reliability and practicality of future investigations.
3. Inadequate user behavior research: The success of webcast marketing hinges on audience behavior; however, the research on audience behavior remains insufficient. Further inquiry is necessary to understand the motives behind audience participation, their decision-making processes, purchasing intentions, and other relevant aspects. Such insights are critical for optimizing marketing strategies and enhancing the overall user experience.

### Corporate brand image

This work on corporate brand image has its roots in marketing and brand management since the 1960s. During this period, companies began recognizing the significance of brand image in consumer purchase decisions and market competition. With the expansion of the global market and intensifying competition, the focus shifted to establishing and managing strong brand images. Over the past few decades, research on corporate brand image has gained prominence as an important academic field. Scholars have explored consumer perceptions and attitudes toward brand image, as well as how companies convey their core values and personality through branding. They have examined the impact of brand identity, brand storytelling, brand experience, and brand communication on brand image. However, certain limitations exist in their research, which can be summarized as follows:

1. Subjectivity and lack of objectivity: Research on corporate brand image often relies on qualitative and quantitative research methods. Qualitative research primarily involves subjective evaluations and opinions of respondents, while quantitative research collects data through methods such as questionnaire surveys. These approaches are susceptible to individual subjectivity, limitations of research samples, and subjective interpretations by researchers, potentially leading to subjective and less objective research findings.
2. Lack of long-term perspective: Research on corporate brand image tends to focus on the current perception and influence of brand image while neglecting long-term development trends. Brand image is a dynamic concept that is continuously influenced by factors such as the market environment, consumer behavior, and competitors. Therefore, research on corporate brand image should incorporate observations and analysis of the evolution and changing trends of brand image. This approach can support companies in formulating long-term brand strategies and plans.
3. Neglect of non-traditional channels and new media: The rise of the Internet and social media has significantly changed brand communication methods and channels. However, some research on corporate brand image still predominantly concentrates on traditional media and channels, overlooking the impact of new media and non-traditional channels on brand image. Therefore, it is crucial to recognize and emphasize the role of emerging media and non-traditional channels in brand image research. This comprehensive understanding enables companies to shape their brand image effectively.
4. Lack of consideration for cultural and regional differences: A company’s brand image may vary across different cultures and regions. However, some research on corporate brand image neglects cultural and regional differences when designing research methods and analyzing results. This oversight may limit the applicability of research findings in diverse cultural and regional contexts. It is essential to consider cultural and regional nuances to ensure the broader relevance of research findings.

Future research on corporate brand image can enhance its validity, objectivity, and relevance by addressing these limitations.

## Webcast marketing

### Analysis of the characteristics of webcast marketing

**Fig 1:**
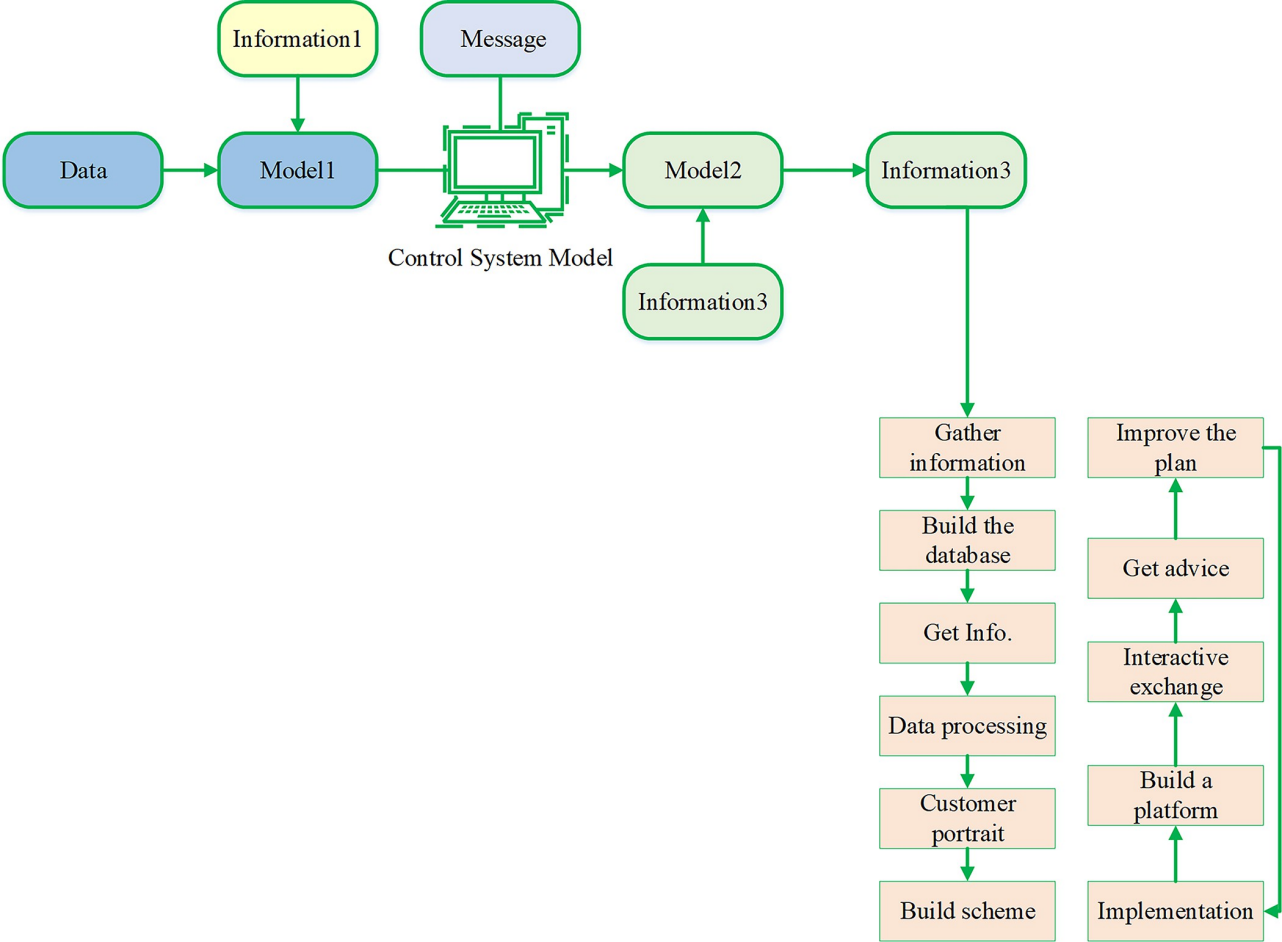
Cosmetics marketing process.

Webcast marketing has emerged as a new avenue for businesses to engage in marketing and promote their brands, capitalizing on the rapid growth of the live broadcast economy [10, 11]. During webcasts, customers have the opportunity to interact directly with the live-stream team by asking questions and making product requests [12–14]. The anchor, in turn, provides feedback and answers questions based on viewer feedback and requests. These interactive activities between anchors and customers help bridge the spatial and temporal gap that exists between businesses and customers in the retail e-commerce environment. Webcast marketing models aim to provide consumers with an experience that closely resembles personal purchasing interactions. Compared to standard e-commerce shopping, webcast marketing offers greater product visibility and authenticity [15, 16]. The host facilitates product perception through product displays, detailed information, and usage scenarios, enabling corporate brands to build relationships with customers and achieve their business objectives. Fig 1 illustrates the cosmetics’ webcast marketing process.

Table 1 presents a comprehensive overview of the distinctions and similarities between the standard online store and the live broadcast online store.

**Table 1:** Comparison of traditional e-commerce model and webcast broadcast e-commerce model.

Comparing content	Traditional e-commerce model	Webcast e-commerce model
**The relationship between products and users**	People search for goods.	Goods wait for people.
**Consumption path**	User-Item	User-Host-Commodity
**Supply chain link**	There are many commodity circulation links, and the cost is relatively high.	Head anchors and institutions can directly connect with the supply chain, effectively shortening the intermediate links of the supply chain and saving the cost of commodity circulation. In addition, anchors and institutions have strong bargaining power and can provide users with products with greater discounts.
**User consumption mode**	Users actively search for products	The anchor mainly recommends products to users
**User consumption needs**	Users have a rigid demand for commodities, and they buy them to meet their material needs. The consumer psychology of users is usually “pay for demand” or “pay for the brand”.	In the case that users do not have a rigid demand for products, the live broadcast e-commerce model creates content marketing through anchors, products, and consumption scenarios, which can stimulate users’ potential consumption needs.

### Customer perceived value

Companies can effectively meet customer needs by understanding their perceptions, and researcher Alexander Zauner has summarized customer perceived value into three stages in an exploratory study. The first stage involves customers evaluating the quality-price relationship to make overall assessments after considering the costs and benefits of practical products. The second stage introduces the theory of “value” in consumer behavior and defines five dimensions of customer perceived value. These dimensions are conceptually independent, meaning that any of these values can influence customers’ decisions depending on different conditions and the specific product or service under consideration. This definition provides clarity to the configuration of customer-perceived value, enhancing its practicality and scientific basis.

In the third stage, customer perceived value is analyzed from dimensions, abstract levels, and model classifications, explaining the applicability of high-level conceptualized customer perceived value to multidimensional conceptualized marketing objectives. From a causal relationship perspective, the different dimensions of customer perceived value are interconnected and can be manifested through certain goods and services. Essentially, they are interchangeable. Considering only the aspect of benefits, customers’ perceived value of a product primarily lies in the ‘trade-off’ between the net benefits gained and the cost differentials incurred.

Customer perceived value is primarily measured through three aspects: functional value, emotional value, and social value, all of which can be effectively demonstrated in webcast marketing. Functional value is observed in how a product’s basic attributes or functions can meet customers’ practical needs in specific areas such as communication and home appliances. In the ‘brand + product launch + webcast’ model of webcast marketing, the webcast content focuses on introducing new products and demonstrating their functionality. Through the webcast, consumers can intuitively observe and learn about the specific features and usage of the products, comprehensively reflecting the functional value in customer perceived value within this model.

Emotional value is mainly derived from how a brand or product meets customers’ emotional needs. Customers are attracted to consumption based on themes, atmospheres, moods, and more. In the ‘brand + influencer + webcast’ model of webcast marketing, everyday people are chosen as live streamers to create a sense of empathy. Consumers easily resonate with the experiences and emotions associated with certain brands and products during the webcast process, satisfying their emotional needs and establishing emotional value towards the brand and products.

Social value is evident when a product represents a customer’s social status, economic strength, and self-realization value, such as high-end watches, luxury handbags, and other luxury goods. In the ‘brand + celebrity + webcast’ model, companies select celebrities that align with the brand’s identity and product positioning as live streamers. Typically, celebrities with higher popularity and social influence are chosen. Consumers watching the live stream are inclined to choose brands and products that match their personal identity and status, driven by their self-value needs and the celebrity effect. The subsequent use of the products allows consumers to display their identity and status, providing additional social recognition and thereby achieving social value.

Most customers choose brands and purchase products and services primarily to satisfy one or several of these values. Therefore, in the webcast marketing model, if a company’s brand and products can meet customers’ preferences or needs, customer-perceived value naturally increases, and establishing customer trust in brand perception becomes relatively easy.

In conclusion, with the emergence of social media marketing, customers have diversified channels to perceive value. By utilizing these social media marketing tools based on customer-perceived value, companies can significantly enhance the possibilities of brand marketing. Furthermore, with the continuous development of the Internet economy, social marketing models are constantly evolving and improving. The emergence of online webcast platforms has introduced a novel approach to social media marketing, making webcast marketing a distinct and innovative subdivision.

### Prediction of sales of cosmetics webcast marketing based on the Back Propagation neural network

This work aims to address the intricate and uncontrollable factors that impact the sales volume of live-streaming e-commerce. To achieve this objective, it introduces artificial neural networks into sales forecasting for online webcasts. Specifically, this work conducts an empirical investigation using cosmetics as a case study. The neural network architecture is designed to encompass the input layer, output layer, and hidden layer neurons. A Back Propagation (BP) neural network model is constructed to forecast sales in online webcasts. Training and optimization tests are carried out with varying numbers of hidden layer neurons. In this empirical study, diverse data related to influencing factors are collected from live-streaming cosmetics and used as input data, while the number of payment orders represents the output data. A BP neural network model is established, and a set of samples is chosen as validation data to empirically evaluate the effectiveness of sales volume forecasting for live-streaming cosmetics.

### Analysis of existing problems in webcast marketing of cosmetics

Cosmetics webcasts, which combine ‘live broadcast + cosmetics,’ have demonstrated the ability to enhance brand awareness and generate substantial revenue. Consequently, numerous cosmetics companies have leveraged the growing trend of webcast marketing by collaborating with Internet celebrities or popular personalities to promote their brands and sell their products during live webcasts. While webcast marketing has the potential to generate significant revenue, it also presents several limitations that can be categorized into three main areas.

1. Immature economic form: Despite facilitating significant economic benefits and contributing to the continuous growth of e-commerce, webcast marketing remains an evolving and developing economic structure [17, 18]. Currently, the predominant form revolves around combining live broadcasting with advertising.
2. Visual appeal-focused marketing model: The marketing approach in webcast marketing predominantly relies on visual appeal. Utilizing celebrities and endorsed brands to promote the effectiveness of cosmetic products aims to captivate the modern mindset of young consumers, who are often influenced by attractive individuals or follow their idols to fulfill their consumption needs, thereby driving high sales.
3. Lack of depth and brand concept: In pursuit of profit, many cosmetics brands neglect the importance of establishing meaningful connections with their audience and assisting them in finding the products that suit their needs. Consequently, these brands often fail to sustain the initial sales enthusiasm following a period of immense success, resulting in the brand losing its relevance and struggling to retain customers.

By addressing these limitations and embracing a more comprehensive approach to webcast marketing, cosmetics brands can establish a stronger foundation for long-term success and customer loyalty.

## Research hypotheses and construction of the theoretical model

### Cognitive matching analysis of the influence of content marketing on brand fit behavior

**Fig 2:**
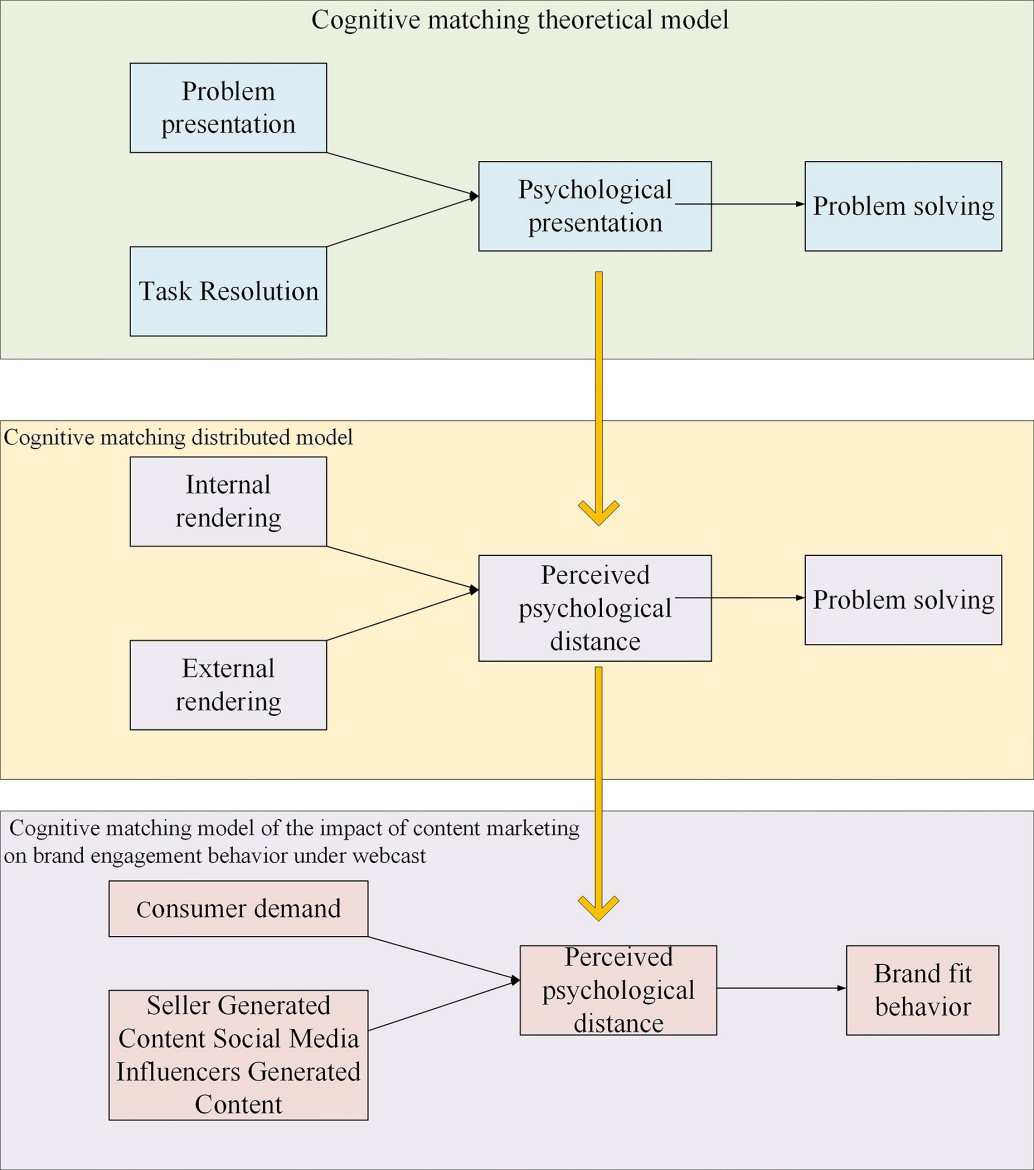
Framework of the cognitive matching theory.

Drawing on cognitive psychology and information processing theory, Vessey (1991) proposed the cognitive matching theory as a research paradigm to investigate individual decision-making processes [19, 20]. The cognitive matching theory elucidates the influence of problem presentation on decision-makers, their cognitive processes, and their preferred approaches to information processing. In the context of webcast marketing, customer requirements can be viewed as internal presentations, while salespeople and social media influencers acting as anchors during live broadcasts can be seen as providers of external presentation information. Consumers exhibit a low perceived psychological distance from the brand, as evidenced by their acceptance of internal and external presentation materials. Consequently, brand-fit behavior ensues. The framework of the cognitive matching theory is illustrated in Fig 2.

### A framework for the impact of content marketing on brand fit behavior under live broadcast

**Fig 3:**
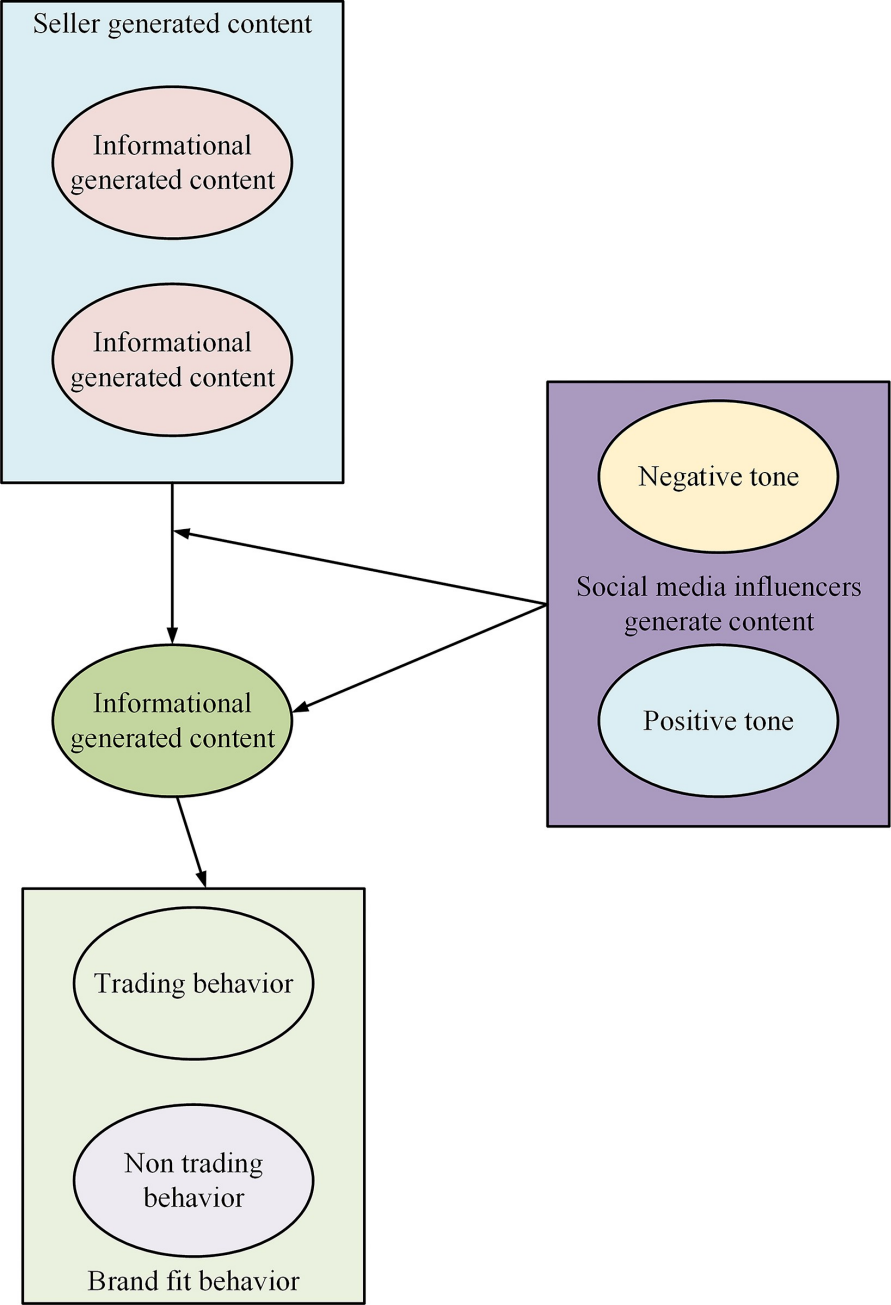
Conceptual model of the impact of content marketing on brand fit behavior under webcasting.

This work examines customer perceptions of psychological distance as a response to emotional states and brand fit behavior as a response to behavioral states. The external environmental stimuli considered in this research comprise seller-generated content characterized by different information features and social media influencer-generated content characterized by different tones [21–23]. Fig 3 depicts the theoretical model.

**Fig 4:**
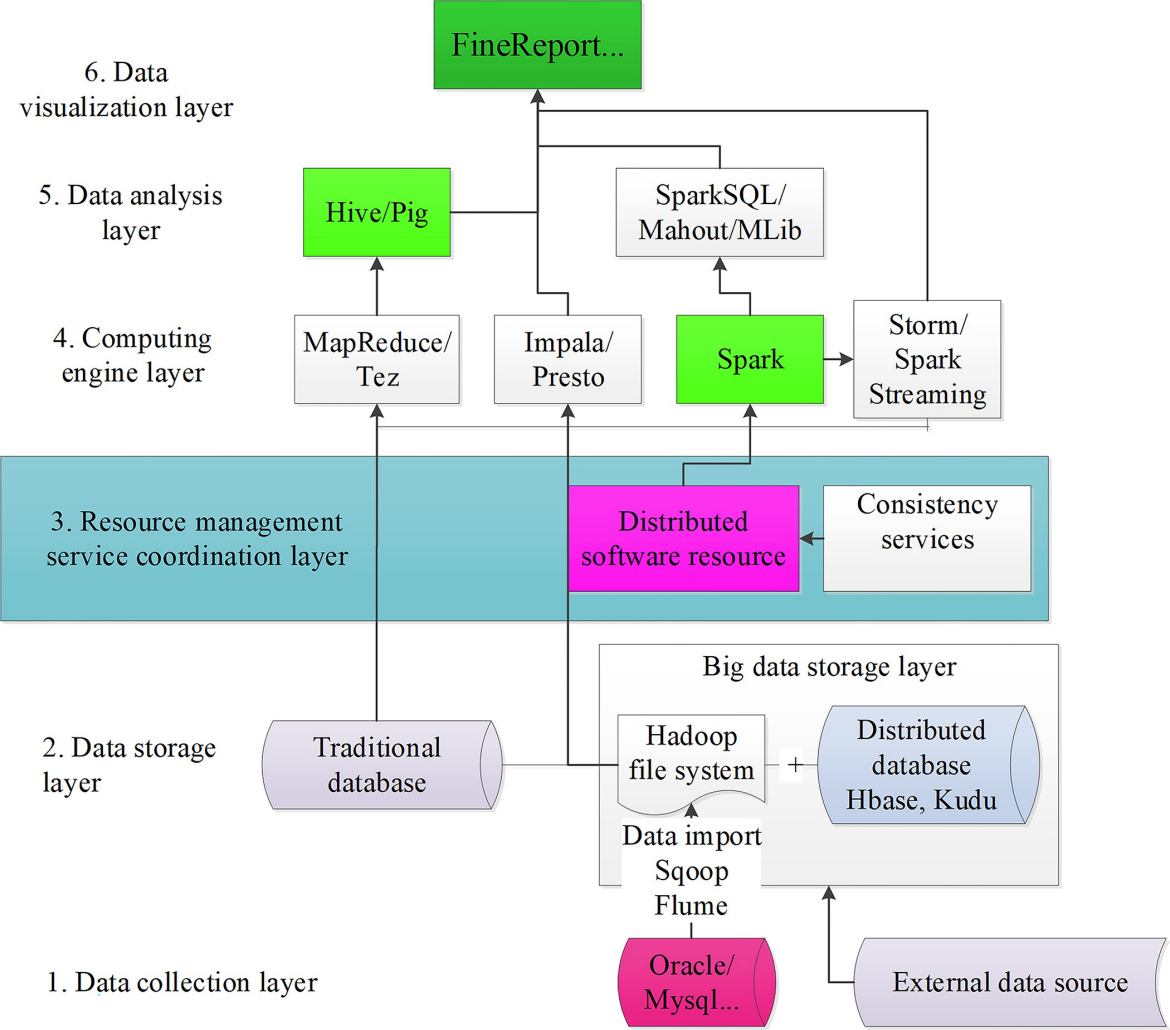
Big data system architecture.

The concept of big data originated from a collaborative effort among several Internet companies in the United States. In contrast to traditional data, big data stands out due to its large volume, variety, rapid input and processing speed, and low data value density. Fig 4 illustrates the architecture of the big data system.

### Research hypotheses

To some extent, webcast marketing strategies can address the limitations of traditional marketing approaches, such as inadequate communication and low engagement. By leveraging webcast marketing, customers’ familiarity and satisfaction with promoted products can be enhanced, reducing the psychological distance between customers and the brand. Moreover, interactive content delivered by brand sellers has the potential to strengthen customers’ positive associations with the brand, consequently narrowing the psychological gap between the brand and its products. For example, brand sellers can actively respond to consumer inquiries and provide additional information through online interactions [24]. Therefore, this work proposes the following hypotheses:

H1a: The interactive content generated by the seller has a positive impact on perceived psychological distance.

H1b: The interactive content generated by social media influencers has a positive impact on perceived psychological distance.

The impersonal nature of online purchasing can create a psychological distance between customers and the brands they purchase, which in turn may impede customer adaptation behavior towards a brand. To address this issue, marketing initiatives that focus on reducing psychological barriers between the brand and the target audience can positively impact customer brand loyalty, repeat purchases, and brand advocacy. Brands hold significant importance for customers, and facilitating brand adaptation can encourage more frequent consumption, ultimately enhancing the brand’s credibility [25]. Therefore, this work proposes the following hypotheses:

H2a: Perceived psychological distance positively impacts brand fit transactional behavior.

H2b: Perceived psychological distance has a positive impact on brand fit non-transactional behavior.

Customers’ perception of the psychological distance between themselves and a brand influences their evaluations and responses toward the brand. This phenomenon, in turn, aids businesses in enhancing product awareness and strengthening customer relationships through social networks [26], thereby expanding the impact of brand marketing. It is important to note that unfavorable content shared by social media influencers can hinder customers from establishing connections with the company and have a detrimental impact on the company. Therefore, this work proposes the following hypotheses:

H3a: Content generated by social media influencers with a positive tone has a positive impact on perceived psychological distance.

H3b: Content generated by social media influencers with a negative tone has a negative impact on perceived psychological distance.

Customers’ growing skepticism towards webcasts has resulted in increased obstacles in online shopping and posed challenges for brands in building connections with them. Social media influencers play a crucial role in effectively promoting brand products to customers to tackle this problem. Influencers can divert customers’ attention toward the promotional content itself, thereby mitigating the impact of their personal characteristics by employing storytelling techniques and adopting unique tones [27].

Therefore, this work proposes the following hypotheses:

H4a: Positive-toned social media influencer-generated content enhances the relationship between seller-informatively-generated content and perceived psychological distance.

H4b: Social media influencer-generated content with a positive tone strengthens the relationship between seller-generated interactive content and perceived psychological distance.

H4c: Social media influencer-generated content with a negative tone weakens the relationship between seller-generated informative content and perceived psychological distance.

**Fig 5:**
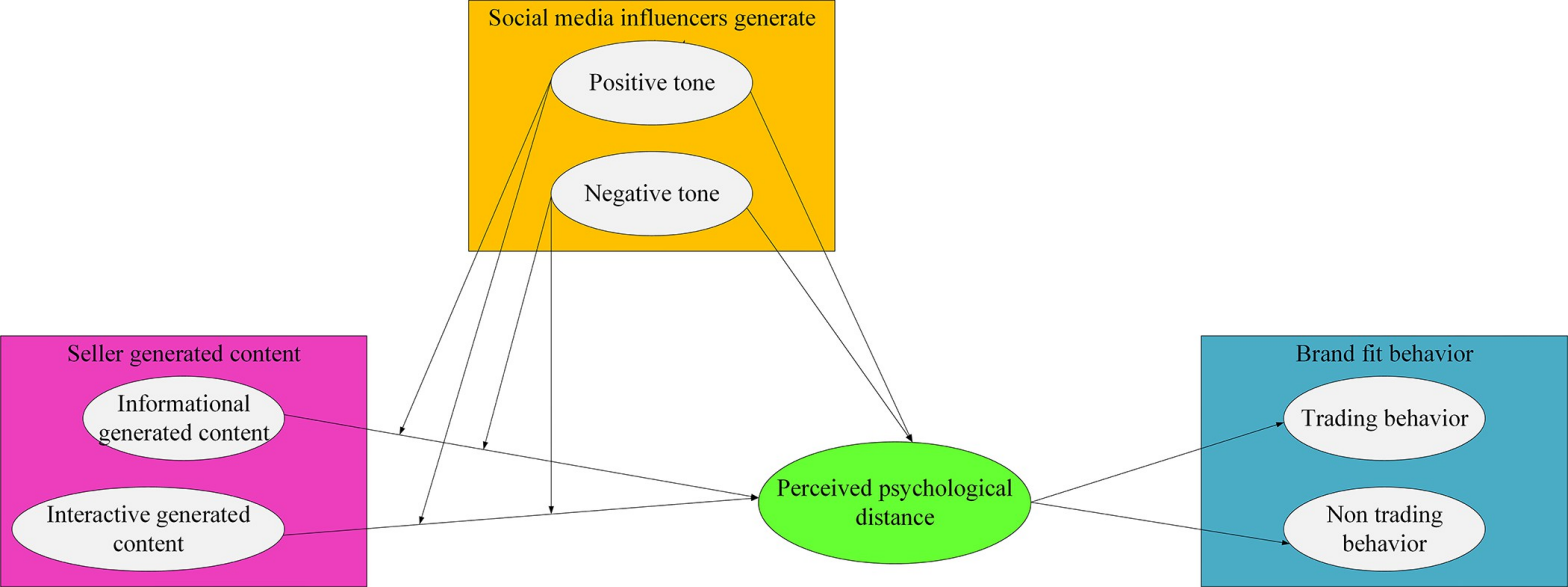
The theoretical model of the influence of content marketing on brand fit behavior under live webcast.

Fig 5 presents the theoretical model of the influence of content marketing on brand fit behavior under live broadcast.

### Study design and data collection

This work examines the influence of content marketing on brand fit behavior in the context of webcasting by utilizing cosmetics sold on the Kuaishou live stream e-commerce platform as a case study. Table 2 summarizes the selected live broadcast sessions on Kuaishou. The choice of cosmetics sold on the Kuaishou e-commerce webcast platform as the research case in this work is motivated by four key reasons. Firstly, the Kuaishou e-commerce webcast platform enjoys immense popularity as one of China’s largest short video platforms, boasting a substantial user base and an active user community. With its distinctive webcast format and social interaction features, this platform has emerged as a prominent player in the e-commerce industry. Thus, an examination of cosmetics sales on the Kuaishou platform can offer valuable insights into current trends in e-commerce webcasts and consumer behaviors.

**Table 2:** Live broadcast sessions on Kuaishou selected for this work.

Serial number	Name	Number of followers	Live time
**1**	Studio-a	87.2 million	January 1st 18:30–24:00; January 3rd 18:30–24:00; January 5th 8:30–24:00;
**2**	Studio-b	55.46 million	January 6, 13:30–18:00; January 7, 13:30–18:00; January 8, 13:30–18:00
**3**	Studio-c	31 million	January 9th 13:30–20:00; January 11th 13:30–20:00; January 13th 13:30–20:00
**4**	Studio-d	13 million	January 14th 18:30–23:00; January 15th 18:30–23:00; January 17th 18:30–23:00
**5**	Studio-e	53 million	18:30–20:00, January 18; 18:30–20:00, January 19; 18:30–20:00, January 20

Secondly, the cosmetics market represents a vast and competitive industry with continually growing consumer demand. The Kuaishou e-commerce webcast platform provides a direct channel for cosmetics brands and sellers to engage with consumers and promote their products. Investigating cosmetics sales on this platform can provide deeper insights into consumer demand, purchasing behavior, and brand loyalty within the cosmetics industry.

Thirdly, the webcast format offers distinct advantages for e-commerce, delivering a more immersive and engaging shopping experience through real-time video demonstrations and interactive communication. Particularly for cosmetics, a webcast allows for the effective showcasing of product usage, the demonstration of effects, and the provision of makeup techniques, thereby capturing consumer interest and influencing purchase intent. Consequently, examining cosmetics sales on the Kuaishou e-commerce webcast platform facilitates an exploration of the role and influencing factors of the webcast format in shaping consumer purchasing decisions.

Lastly, the Kuaishou e-commerce webcast platform places significant emphasis on social interaction, enabling consumers to actively engage with hosts and other viewers through bullet comments, comments, and interactive features [28]. This social interaction plays a pivotal role in establishing consumer brand identification and fostering loyalty. Studying cosmetics sales on the Kuaishou platform makes it possible to investigate the impact of social interaction on consumer purchasing decisions and brand perception.

In conclusion, selecting cosmetics sold on the Kuaishou e-commerce webcast platform as a research case provides valuable insights into e-commerce webcast trends, consumer purchasing behavior, and brand influence within the cosmetics market.

This work opts for the Kuaishou live e-commerce platform as the research backdrop and beauty products as the focal subject of analysis to examine the influence of content marketing on brand congruence behavior within the realm of online live streaming. The dataset encompasses the subsequent primary constituents: Live streaming platform data encapsulates parameters pertinent to live streaming technology, network performance data within the 6G environment, and the temporal extent of live streaming sessions. User interaction data comprises metrics such as user viewing duration, likes, comments, and sharing activities [29]. Brand content data involves live streaming content linked to the brand, content classifications, and imaginative expressions. To ensure the fidelity and precision of the data, rigorous cleansing, arrangement, and de-identification protocols were executed. In the course of these procedures, any sensitive information was expunged to uphold user and brand confidentiality [30]. Table 2 furnishes a comprehensive snapshot of the selected Kuaishou live-streaming sessions that were the focus of this investigation.

## Empirical analysis of content marketing impact model on brand fit behavior

### Data analysis of structural equations

This work investigates the impact of content marketing on brand fit behavior in the context of webcasts, focusing on the raw material of live clips from the Racer live e-commerce platform. Table 3 presents the results of the path model fitting index for the model.

**Table 3:** Structural equation model fitting index.

Index	*df*	*p*	Chi-square degrees of freedom ratio *x*^2^/*df*	Goodness of fit index	Root mean square error of approximation	Comparative fit index
**Standard**	/	>0.05	<3	>0.85	<0.10	>0.9
**value**	28.1	0.060	2.21	0.90	0.081	0.922

### Structural equation modeling analysis

**Fig 6:**
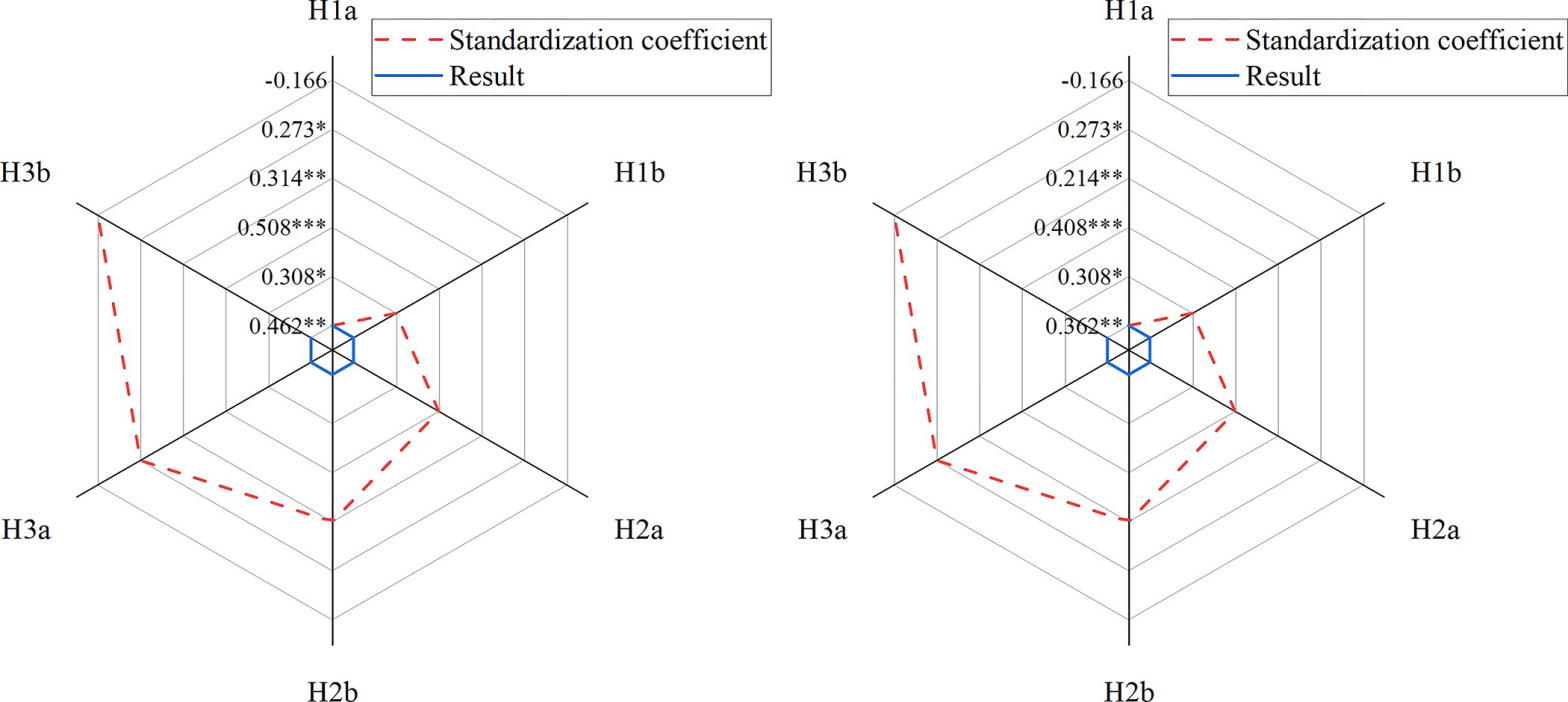
Model assumption path coefficients and results (a. First test; b. Second test). In Fig 6 *, **, and *** denote " *p*<0.05, *p*<0.01, and *p*<0.001, respectively.

A structural equation model is employed to examine the relationships among various factors and their impacts on each other. This work specifically investigates the relationships between user-generated content and psychological distance, user-generated content and brand fit behavior, and media-generated content and psychological distance. Fig 6 illustrates the results of the path coefficient analysis, providing insights into the precise relationships between these variables.

Fig 6 illustrates the findings regarding the relationships between different variables. The results provide support for H1a, indicating that informational content significantly enhances the sense of psychological distance. Additionally, H1b is supported, as interactive user-generated content has a substantial positive impact on psychological distance. Furthermore, this work confirms the validity of H2a, which suggests that psychological distance has a significant and positive influence on brand fit transaction behavior. Similarly, H2b is supported, as increased psychological distance positively affects both transactional and non-transactional brand fit behavior. Moreover, the results provide evidence in favor of H3a, demonstrating that user-generated content on social media platforms strongly affects the perception of proximity. Conversely, H3b is substantiated, indicating that content generated through social media with a negative tone has a notable detrimental effect on perceived psychological distance.

### Mediating effect test

This work examines the differential impact of information-based sellers’ and interactive sellers’ content during live broadcasts, aiming to explore the moderating role of customers’ perceived psychological distance from businesses. Table 4 lists the specific results of the path coefficient analysis.

**Table 4:** Model hypothesis path coefficients and results.

environment							
5G				6G			
Path		Standardized coefficient	Test results	Path		Standardized coefficient	Test results
Seller-generated information	Brand fit transaction behavior	0.159*	significantly	Seller-generated information	Brand fit transaction behavior	0.179*	Significantly
Seller-generated information	Brand fit non-transactional behavior	0.341**		Seller-generated information	Brand fit non-transactional behavior	0.381**	
Seller-generated information	Brand fit transaction behavior	0.231**		Seller-generated information	Brand fit transaction behavior	0.241**	
Seller-generated interactive information	Brand fit non-transactional behavior	0.391*		Seller-generated interactive information	Brand fit non-transactional behavior	0.401*	

Table 4 illustrates the mediating role of the seller’s perceived psychological distance in the relationship between seller-generated information and the buyer’s brand-appropriate purchase decisions in the 5G environment. Notably, the seller’s psychological distance significantly mediates the connection between user-generated content and brand-aligned purchasing behavior. Moreover, the buyer’s perception of the seller’s psychological distance moderates the seller’s interactive content, influencing the buyer’s brand-appropriate non-transactional behavior. The intermediary effect of type-generated content on brand-fit purchasing behavior is substantial. Perceived psychological distance mediates the relationship between seller-generated information and the consumer’s non-transactional brand-fit behavior. It also substantially mediates between seller-interactively generated content and brand-fit transactional behavior, which is consistent in both the 5G and 6G environments. In the 5G environment, the active content generated by the webcast marketing platform positively impacts brand fit (β = 0.46, p<0.01; β = 0.31, p<0.05). Additionally, the high traffic transmission rate of the webcast marketing platform in the 6G network environment may positively influence brand fit (β = 0.51, p<0.001). The positive moderating effect of interactive generated content is also significant (β = 0.42, p<0.01; β = 0.02, p<0.001).

### Validation of the feasibility of optimizing the webcast marketing platform by predicting sales

**Fig 7:**
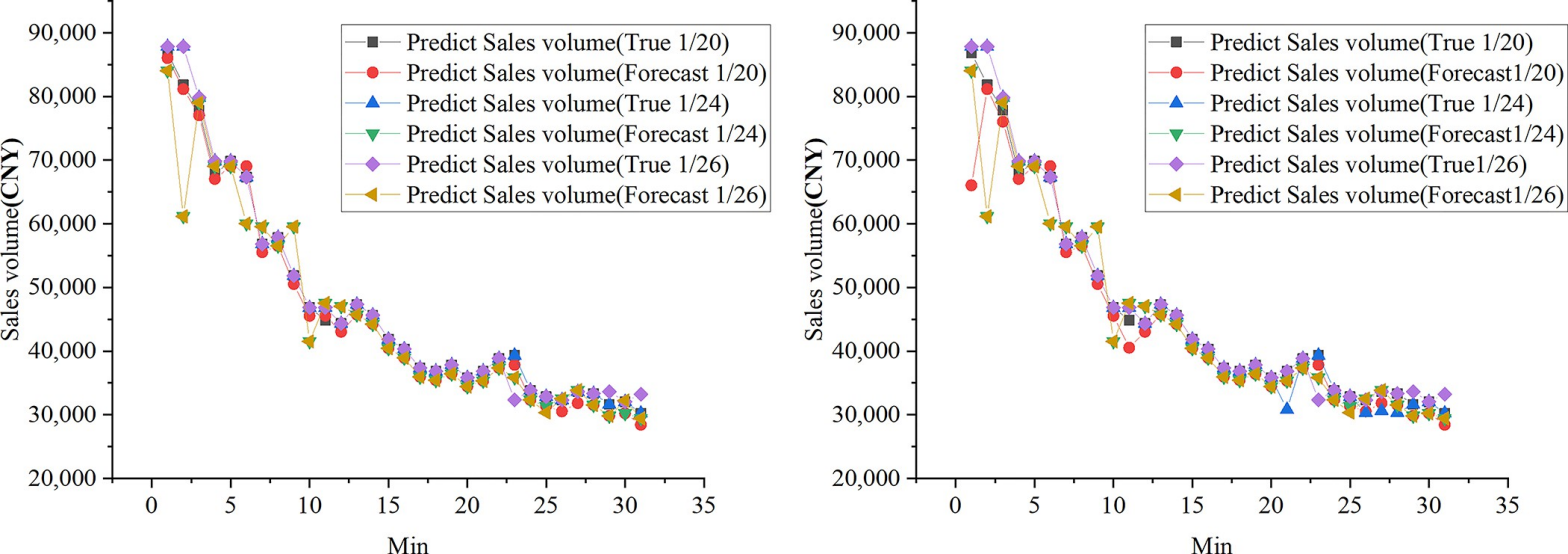
Comparison of forecasted live broadcast sales and target sales (a. Studio-a; b. Studio-b).

The proposed optimized webcast marketing plan is incorporated as an input variable in a conventional forecasting model to predict the sales of three upcoming live broadcasts on the platform. Fig 7 illustrates the comparison between the projected sales and the desired sales for a specific live broadcast.

Fig 7 illustrates the projected sales and actual sales for live broadcasts that were yet to take place on January 20, 2022. For Studio-a, the predicted earnings were 1,451,718.6 CNY, while the actual earnings amounted to 1,440,029.8 CNY, resulting in a relative error of only 2.6%. Similarly, Studio-b was projected to achieve sales of 1,251,718.6 CNY, and the actual sales reached 1,214,029.8 CNY, with a relative inaccuracy of just 2.3%. These data demonstrate the webcast marketing platform’s ability to meet the marketing needs of certain brands.

### Correlation analysis of incentive constraint and performance

**Fig 8:**
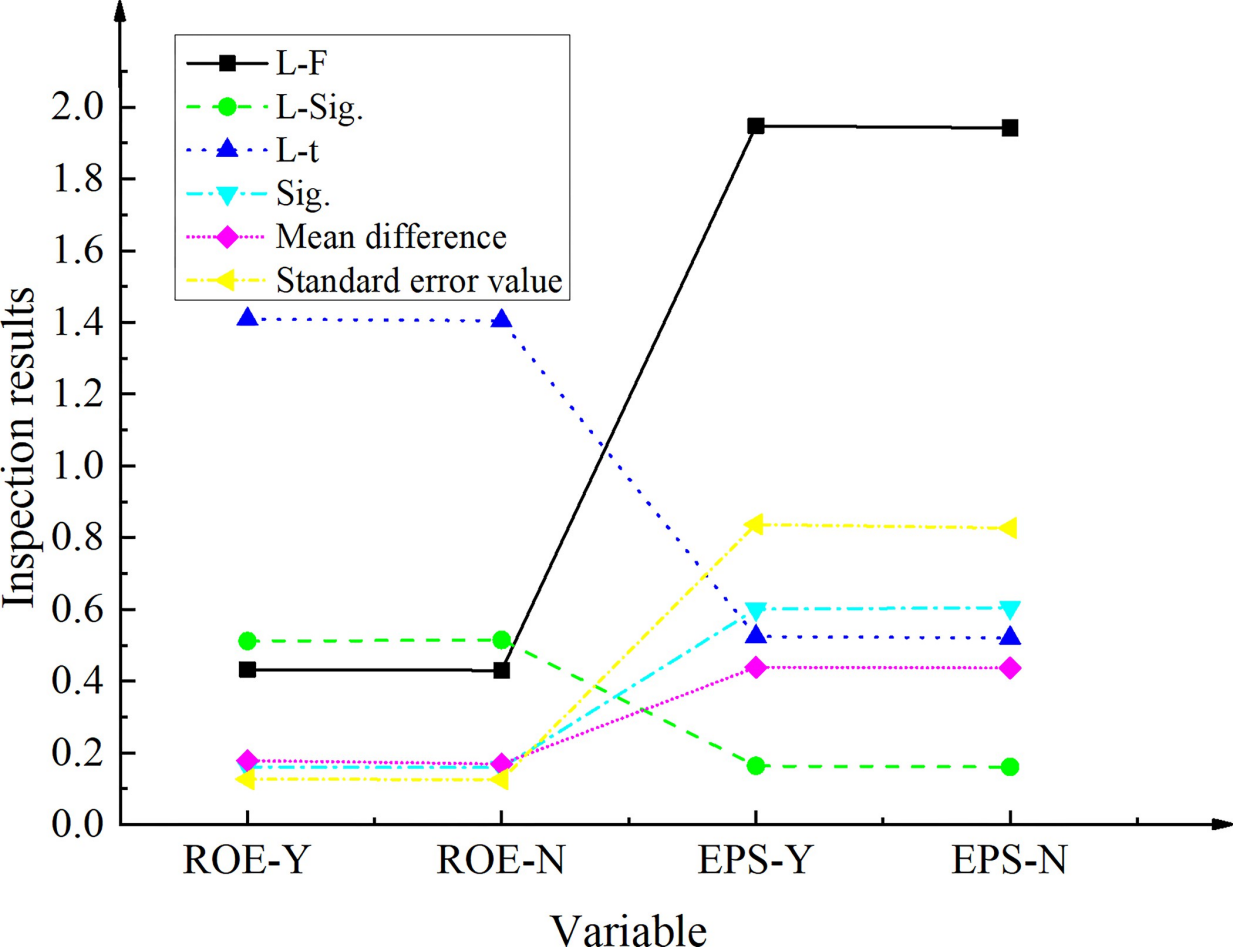
Test results of independent samples of study enterprises (L- prefix represents Levene test; ROE denotes the Return on Equity; EPS refers to Earnings Per Share; ROE-Y means that the variances are equal; ROE-N means that the variances are not equal).

This work gathers data from the Shenzhen Guotaian database and the Juchao Information Website to survey a total of one hundred organizations intending to adopt webcast marketing by 2022. As a control group, one hundred businesses that have not yet employed live marketing were selected, with this work and comparison samples divided into Groups A and B, respectively. The analysis focuses on optimizing the webcast platform, anchor performance, and creating brand content. Fig 8 presents the findings obtained from independent samples of research companies.

The Levene test was conducted to examine the variance equation, revealing an F-value of 0.431 and a Sig. value of 0.513 for ROE, as well as an F-value of 1.949 and a Sig. value of 0.165 for EPS. In both cases, the variables showed significance levels greater than 0.05. Consequently, there were no significant differences observed between the study samples in Group A and Group B. Moreover, the Sig. values for both ROE and EPS were 0.162 and 0.602, respectively, indicating that the variance was equal between the two groups. Both significance levels were greater than 0.05, further supporting the lack of difference between Group A and Group B in terms of their mean values for ROE and EPS.

**Fig 9:**
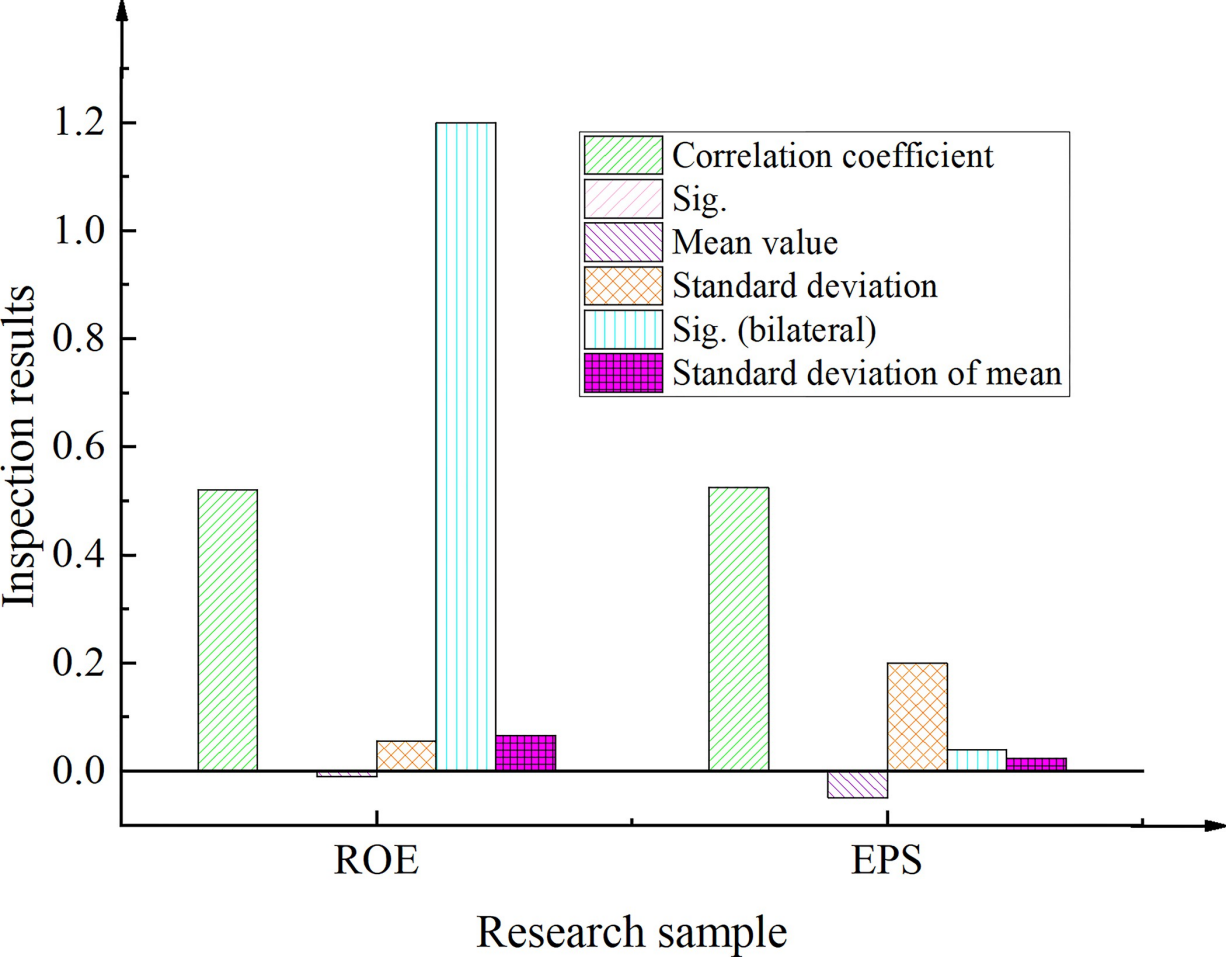
The sample test results of the research samples A and B after matching.

Fig 9 presents a comparative analysis of the firm’s performance before and after the implementation of live marketing for the samples in groups A and B.

Fig 9 illustrates the results of the paired-sample correlation analysis for the period 2022–2023. The Sig. value associated with this analysis is found to be less than 0.05, indicating a lack of statistical significance. Specifically, the mean value of the paired difference corresponding to ROE is calculated as -0.01033, while the corresponding value for probability is 0.13, which is higher than the significance level of 0.05. In addition, the average pairwise difference for EPS is determined to be -0.04912, which is below the threshold of 0.05. This result suggests a significant increase in EPS between 2022 and 2023. These findings provide evidence of the positive impact of live marketing on optimizing business performance, particularly in terms of EPS.

## Discussion

The experimental findings indicate that the network webcast marketing platform, leveraging 6G development, significantly influences the optimization of brand content creation and marketing strategies. Firstly, the adoption of high-speed and low-latency communication technology ensures smoother and real-time webcasts, enabling the audience to enjoy higher-quality video content. This enhanced streaming capability empowers brands to effectively showcase their products and services, providing a more immersive and captivating brand experience, thus fostering increased audience engagement and interest. Secondly, the ability for cross-device and cross-platform interaction introduces new possibilities for brand content creation. The audience can interact with brands through various devices and platforms, such as real-time comments, likes, and shares. Brands can promptly respond to audience feedback and demands, making real-time adjustments and personalized content creations that align with audience preferences. This dynamic approach enhances audience engagement and strengthens brand identification. Furthermore, the webcast platform based on 6G technology offers brands diversified communication channels, including augmented reality and virtual reality technologies. By leveraging these innovative communication methods, brands can create more immersive and enriched brand experiences for the audience. Interacting with the virtual world allows the audience to better understand product features, experience services, and establish a closer relationship with the brand. Additionally, data-driven marketing strategies become more effective on the 6G webcast platform. The platform collects abundant real-time data, providing brands with in-depth insights into audience behavior patterns, preferences, and demands. This enables more precise targeting and personalized content creation. By analyzing the data, brands can better anticipate market trends, formulate targeted marketing strategies, and enhance brand awareness and loyalty among the audience. In conclusion, the experimental results of the network webcast marketing platform based on 6G development demonstrate its positive impact on optimizing brand content creation and marketing strategies. This advancement offers brands innovative, interactive, and personalized opportunities, fostering interaction and engagement between brands and the audience. Consequently, it enhances brand influence, recognition, and market competitiveness.

Theoretically, this work delves into the potential of optimizing webcast marketing platforms through 6G development and examines the impact of such optimization on-brand content creation. It presents a novel perspective for comprehending and leveraging the future possibilities of 6G technology in the marketing domain. By investigating the application of 6G in webcast marketing, a deeper understanding of the influence of 6G technology on brand marketing strategies and user experiences can be attained, providing a solid theoretical foundation for future research and practical implementation. Practically, this work offers valuable insights for businesses and marketing practitioners. Firstly, businesses can enhance the effectiveness and appeal of brand content creation by optimizing webcast marketing platforms and integrating innovative 6G technology applications. This improvement enables businesses to differentiate themselves in a competitive market, attract a larger target audience, and augment brand visibility and reputation. Secondly, this work underscores the pivotal role of brand content creation in webcast marketing. By comprehending the impact of 6G technology on content creation, businesses can leverage innovative technological means and strategies to design and disseminate brand content, thereby achieving higher levels of brand attention and user engagement. Additionally, this work emphasizes the significance of businesses staying abreast of future development trends and application prospects of 6G technology. With the continuous advancement of 6G technology, webcast marketing will witness further innovation and transformation, necessitating businesses to proactively monitor and adapt to these changes to maintain a competitive edge. In summary, this work contributes to the understanding and utilization of 6G technology in optimizing webcast marketing platforms. It sheds light on the impact of 6G on-brand content creation, offering valuable insights for businesses and marketing practitioners. Furthermore, it underscores the importance of keeping abreast of future developments in 6G technology to remain competitive in the dynamic landscape of webcast marketing.

When comparing the findings of this work with the research conducted by Rakshit et al. (2022) [29], it is evident that the conclusions of this work indicate that in the context of a 6G network environment, a webcast marketing platform with high data transmission rates may enhance brand fit, and the content generated by the webcast platform has a significant positive moderating effect on information-based and user-generated content. On the other hand, Rakshit et al.’s research emphasizes the positive influence of activity content generated by webcast marketing platforms on brand fit but does not mention the moderating effect of content on information-based and user-generated content. The research findings of this work mention the moderating effect of content on information-based and user-generated content in the conclusions, indicating a more comprehensive and detailed exploration of the relationships between different factors and richer research outcomes in this regard. When comparing the research findings of this work with those of Xu and Kong (2021) [30], the results are presented in Table 5.

**Table 5:** Error verification of the prediction results via the BP neural network.

Date	Number of original payment orders (units)	Prediction results via the BP neural network	Research findings by researchers Xu and Kong
**1**	167	163	151
**2**	146	141	132
**3**	70	66	56

According to the findings presented in Table 5, the algorithm reported here outperforms the algorithm proposed by Xu and Kong in terms of predicting the original payment order quantity. Specifically, the proposed algorithm incorporates a more precise and effective model or technique for accurate prediction. It encompasses a broader range of features or employs more sophisticated algorithms to capture the pertinent factors that influence the order quantity, resulting in more precise predictions. Furthermore, this algorithm adopts optimized approaches in data processing, feature selection, and model training, thereby achieving a higher level of accuracy.

## Conclusion

Webcast marketing has emerged as a prominent advertising method, gaining popularity among businesses due to its accessibility, cost-effectiveness, quick results, and precise targeting capabilities. It represents a new marketing tool that has evolved alongside technological advancements in the modern era. Webcast marketing enables brands to achieve high-efficiency results at a low cost by leveraging pinpoint marketing, big data technologies, interactivity, and measurable performance. The growth of webcasting technology has led to the proliferation of live stream services, prompting the need to analyze and research the best webcast marketing practices to attain desired communication outcomes. This work examines the current landscape of cosmetic webcast marketing platforms and develops a theoretical framework to evaluate the impact of the webcast marketing platform experience on brand fit. Additionally, several hypotheses are proposed to explore the relationship between participation in a live marketing platform and business success. The empirical analysis is conducted in the context of 5G to test these hypotheses. The findings of this work demonstrate the following key results: Firstly, the active content generated through the webcast marketing platform positively influences brand fit (β = 0.46, p<0.01; β = 0.31, p<0.05). Secondly, in a 6G network environment, there is a significant positive correlation between the transmission rate of the webcast marketing platform and brand fit (β = 0.51, p<0.001). Lastly, the content generated by the webcast marketing platform exerts a significant positive regulatory effect on information-based and interactive generated content (β = 0.42, p<0.01; β = 0.02, p<0.001). In conclusion, webcast marketing presents a promising avenue for businesses to achieve their marketing objectives. This work’s findings highlight the positive impact of active content and transmission rate on brand fit, underscoring the importance of utilizing webcast marketing platforms effectively. By generating engaging and interactive content, businesses can enhance their brand fit and capitalize on the advantages offered by webcast marketing. These insights contribute to the understanding of the role of webcast marketing in driving brand success, particularly in the context of 6G networks.

The work presents several limitations that should be acknowledged. Firstly, while the current status and issues of the webcast marketing platform are analyzed, there is a lack of in-depth exploration regarding specific methods and implementation strategies to address these problems. The optimization solutions provided lack detailed explanations on how to apply sixth-generation communication technology to improve the platform’s operations and functionalities. Secondly, although a conceptual framework is constructed for the relationship between the webcast marketing platform experience and brand fit, along with the proposal of relevant hypotheses, there is no clear indication of the specific methods and indicators for measuring and evaluating these concepts and relationships. Furthermore, only empirical analysis in the context of 5G is considered when validating the hypotheses, lacking actual verification in a 6G environment.

Future studies should consider the following aspects:

1. Elaborate on the optimization solutions by providing more specific methods and implementation strategies to address the current issues of the webcast marketing platform. Explore how advanced communication technologies can be leveraged to improve platform performance and functionality in conjunction with the development status of sixth-generation communication technology.
2. Develop measurement and evaluation methods to establish reliable metrics and evaluation criteria for the relationship between the webcast marketing platform experience and brand fit. Quantify and validate the correlation between these concepts through accurate indicators and robust data collection methods, thereby enhancing the credibility and practicality of the research.
3. Consider verification in a 6G environment by extending the validation results from the context of 5G to a 6G background, comprehensively evaluating the performance and impact of the webcast marketing platform under different communication environments. Take into account the characteristics and technologies of 6G to further explore the relationship between the webcast marketing platform and brand fit.

Through in-depth research in these areas, a better understanding of the success factors of the webcast marketing platform can be achieved, leading to more specific and feasible solutions to improve platform performance and generate new brand content.

## Supporting information

S1 Data (ZIP)
