# Supplementary material for: Webcast marketing platform optimization via 6G R&D and the impact on brand content creation
Source: PLoS One. 2023 Oct 19;18(10):e0292394. doi: 10.1371/journal.pone.0292394 (PMC10586639; doi:10.1371/journal.pone.0292394)
Supplement: S1 Data — (ZIP) [file pone.0292394.s001.zip › data packet/Code Description.docx]

The purpose of this code is to predict the brand impact using a trained linear regression model and visualize the relationship between different features and brand impact.

**1. Brand Impact Prediction Section:**

- Firstly, an array named `sample_input` is defined, containing three feature values: `6GUsage`, `LiveStreamingDuration`, and `PlatformEngagement`.

- Next, the trained model `model` is used to predict the brand impact for `sample_input`, and the predicted impact value is stored in the variable `predicted_impact`.

- Finally, the code uses a `print` statement to display the predicted brand impact value, rounded to two decimal places.

**2. Feature vs Brand Impact Visualization Section:**

- Firstly, a scatter plot is created, where the x-axis represents the feature values and the y-axis represents the actual brand impact values. This scatter plot showcases the distribution of feature values against the actual brand impact.

- Next, a regression line is plotted using the `plot` function. This line represents the model's predicted results within the current range of feature values and is depicted in red.

- The `xlabel` and `ylabel` functions are used to set labels for the x-axis and y-axis respectively. The `legend` function adds a legend to distinguish between the actual data points and the regression line.

- The `title` function sets the title of the chart, providing a clear explanation of the relationship between the feature and brand impact.

The effect of this code is to demonstrate the predicted brand impact values by the model under different feature value scenarios. It contrasts the actual data points with the regression line. Additionally, the code illustrates how to create multiple charts to visualize the relationship between different features and brand impact, enhancing the understanding of the impact of various features.

**3. Other Feature vs Brand Impact Visualization Section:**

- Firstly, a large figure is created using `plt.figure(figsize=(12, 4))` to accommodate two subplots.

- `plt.subplot(1, 2, 1)` is used to create the first subplot. The `plt.scatter` function is employed to create a scatter plot depicting the relationship between the `LiveStreamingDuration` feature values and the actual brand impact.

- A regression line is plotted using `plt.plot`, with the predicted relationship represented in red.

- `plt.xlabel` and `plt.ylabel` set the labels for the x-axis and y-axis, and `plt.legend` adds a legend.

- `plt.title` assigns a title to the subplot, describing the relationship between the feature and brand impact.

- Next, `plt.subplot(1, 2, 2)` is used to create the second subplot, and a similar procedure is followed to plot the relationship between `PlatformEngagement` feature values and brand impact.

- Finally, `plt.tight_layout()` is utilized to ensure an appropriate layout for the subplots, preventing overlap.
